# Intussusception

**Published:** 1872-03-01

**Authors:** Cyrus C. Reichard

**Affiliations:** Mitchellville, Iowa


### INTUSSUSCEPTION.

BY CYRUS C. REICHARD, M. D., MITCHELLVILLE, IOWA.

The great mortality attending this disease, and its comparatively rare occurrence, have induced me to report the following case:

Sept. 19, 1871.—I was called at 8 a. m. to see E. J.V., aged 32, of sanguine temperament, healthy habits—American. He is a farmer, energetic and industrious. The day preceding his attack was Sunday, and while visiting a neighbor he ate heartily of grapes, apples and walnuts; all through the following night he suffered with colicky pains. When I arrived he was perspiring profusely from intense pain of a paroxysmal character. Being very busy at the time, and, upon inquiry, finding he had had a discharge from the bowels about two hours before my arrival, I hastily diagnosed the disease colic, and promptly mitigated the pain by injecting morph. sulph. gr. ¼, hypodermically. I then ordered a cathartic, and leaving some morphia powders to be taken at intervals through the day, I continued my ride.

I was recalled the same day at 6 p. m., the messenger stating that the pain had returned and he was vomiting. I found him vomiting persistently; pulse full and frequent, and an anxious, appealing expression of countenance. Making a careful examination of what *he* designated the "painful spot" I discovered a tumor, with its inferior margin at the junction of the small with the large intestine, and extending to a point to the right of and opposite the umbilicus. It was not, at this time, very tender to the touch. Percussion elicited marked dullness over the ileo-cœcal valve. The stethescope placed over the tumor gave negative results, but on either side a gurgling sound was plainly heard. In view of the hyper-irritability of the stomach I gave, as a cathartic, hydrarg. chlo. mit. and bis. sub. nit., which was retained several hours with no perceptible effect on the bowels. I then repeated the dose, ordered anodyne fomentations to the abdomen, and administered copious enemata of warm water.

Sept. 20, 8 a. m.—The purgatives given yesterday have had no effect—at least no *good* effect—and the vomiting has become stercoraceous. The pain is still very severe, recurring at short intervals, and confined to the same spot; I relieve it by hypodermic injection of morphia. Have repeated the injections per rectum, and kept the abdomen covered with anodyne fomentations. Have not given any purgative to-day as they seemed to add to his distress yesterday by inverting the peristaltic wave and promoting vomiting.

21st.—Countenance anxious and sunken; pulse small and frequent; has had but little rest; thinks there is no hope for him; still vomits large quantities of fluid mixed with fecal matter. Called in consultation my friend, Dr. Simonton, of Newton, who arrived at 3 p. m. After making a thorough subjective and objective examination, he agreed with me as to the nature of the lesion—which was gratifying, as I have great respect for his diagnostic abilities. To disinvaginate the incarcerated bowel we adopted the method first practiced by Dr. Tate, of Georgia, viz.: generating air within the body. We first overcame muscular opposition by anæsthetizing the patient fully, and having injected a half gallon of water, we introduced sixty grains of bi-carbonate of soda, dissolved in three ounces of water, which was immediately followed by a tartaric acid solution of like strength and quantity—the acid solution was introduced *very slowly* and *cautiously*, lest by a too rapid and powerful an inflation the integrity of the intestines would be jeopardized. After the distension had been carried to a limit consistent with safety, we kept it up at least ten minutes, while we gently kneaded the abdomen at the point of strangulation. After the expulsion of the water and air (midnight) the tumor had disappeared, also the pain, and he rested quite well till morning, when he had a discharge that we were convinced came from above the point of invagination, as it contained some half dozen or more grape seeds, which could only have lodged in the folds of constricted intestines. From this time very little treatment was needed except to check a rather obstinate diarrhœa which I looked upon as the result of prostration of the vaso-motor nervous system, and which was readily overcome by the use of strychnia, iron and opium.

The patient says he has had as good health since as before his attack.

Remarks.—My opportunities for research are limited, but as far as I have been able to learn anæsthetics are not generally given in these cases. They seem, in my estimation, to meet two of the indications exactly—they abolish the inherent contractility of the muscular fibre—the great antagonist of distension—and relieve the pain attending inflation, which must be very severe. Notwithstanding I favor their use I am convinced peculiar care should be exercised in their administration, in cases where the "inflation" treatment is employed, on account of the pressure exerted in the thoracic viscera. Ether is preferable to chloroform because less dangerous.

Mitchellville, Iowa, Feb. 21, 1872.

